# Exploring Factors Affecting Emergency Medical Services Staffs' Decision about Transporting Medical Patients to Medical Facilities

**DOI:** 10.1155/2014/215329

**Published:** 2014-05-07

**Authors:** Abbasali Ebrahimian, Hesam Seyedin, Roohangiz Jamshidi-Orak, Gholamreza Masoumi

**Affiliations:** ^1^Department of Health Services Management, School of Health Management and Information Sciences, Iran University of Medical Sciences, No. 6, Rashid Yasemi Street, Vali-e-asr Avenue, Tehran 1995614111, Iran; ^2^School of Health Management and Information Sciences, Iran University of Medical Sciences, Tehran, Iran; ^3^Department of Emergency Medicine, School of Medicine, Iran University of Medical Sciences, Tehran, Iran

## Abstract

Transfer of patients in medical emergency situations is one of the most important missions of emergency medical service (EMS) staffs. So this study was performed to explore affecting factors in EMS staffs' decision during transporting of patients in medical situations to medical facilities. The participants in this qualitative study consisted of 18 EMS staffs working in prehospital care facilities in Tehran, Iran. Data were gathered through semistructured interviews. The data were analyzed using a content analysis approach. The data analysis revealed the following theme: “degree of perceived risk in EMS staffs and their patients.” This theme consisted of two main categories: (1) patient's condition' and (2) the context of the EMS mission'. The patent's condition category emerged from “physical health statuses,” “socioeconomic statuses,” and “cultural background” subcategories. The context of the EMS mission also emerged from two subcategories of “characteristics of the mission” and EMS staffs characteristics'. EMS system managers can consider adequate technical, informational, financial, educational, and emotional supports to facilitate the decision making of their staffs. Also, development of an effective and user-friendly checklist and scoring system was recommended for quick and easy recognition of patients' needs for transportation in a prehospital situation.

## 1. Introduction

Ambulance service is at the forefront of healthcare services [1]. During the past decade, the need for ambulance service and patient transportation by emergency medical services (EMS) has increased significantly. For example, in England, this increase has been about 16% [2]. In our country, Iran, about 80–85% of all EMS missions are performed for providing prehospital care to patients with medical problems and transporting them to medical facilities [3, 4]. However, not all of these transportations are really urgent. Evidence shows that a large number of EMS missions performed in Iran, the United States of America, and Great Britain are dispensable [3–6]. Knapp et al. also noted that inappropriate and excessive use of EMS is a common problem faced by prehospital care providers [7]. EMS misuse expands EMS staffs' workload, imposes a significant financial burden on the prehospital care system [8], and increases the rate of undue hospital admissions [9]. Consequently, developing an effective control system for determining patients who really need transportation is of the utmost urgency. A basic prerequisite for developing such a system is to determine “who” really needs ambulance services, “why” he/she needs such services, and “where” he/she is located [10].

In many countries, determining patients' need for transportation is up to EMS staffs. They have the opportunity to visit, assess, and provide care to patients in prehospital settings and make decisions about their need for transportation [11, 12]. Consequently, EMS staffs have a unique status for identifying patients' real need for transportation [11]. However, research findings do not support the accuracy of all the judgments and decisions made by EMS staffs about patients' need for transportation [8, 11, 12]. For example, Mann and Guly invited two groups of emergency and family physicians to determine the need for transportation among seventeen patients hospitalized in the emergency ward. They found that the physicians of the two groups had consensus only over 20% of cases [5]. Ebrahimian and Khalesi also found that EMS workers and emergency physicians' agreement on the need of 798 medical patients for transportation was equal to 50.14% [4]. In another study, Challen and Walter found that only 65% of all 215 patients who had been transported to hospital by EMS needed hospitalization [2]. Regarding these disagreements, Fullerton et al. noted that transportation-related judgments and decisions are made mainly based on mental processes and the context of the emergency situation, rather than scientific evidence [12]. This is mainly due to the fact that EMS staffs have different levels of knowledge, expertise, and clinical judgment and decision-making abilities [4, 12]. On the other hand, there is no clear accepted definition for “medical necessity” [8]. Accordingly, deciding about the accuracy of EMS staffs' clinical judgments and decisions is increasingly difficult. Moreover, although the majority of EMS missions are performed for transporting medical patients, predictors of need among these patients for transportation are poorly known [3, 4]. Consequently, exploring EMS staffs' transportation experiences and criteria seems crucial. We conducted this study to reduce this gap. The aim of this study was to explore factors affecting EMS staffs' decision about transporting medical patients to medical facilities.

## 2. Methods

### 2.1. Design

This was a qualitative study conducted by using the qualitative content analysis approach. Content analysis is an analytic approach and a scientific method that provides new insight and better understanding about the intended phenomena [13]. This study was conducted in 2013.

### 2.2. Participants

The study population consisted of all EMS staffs working in prehospital care facilities located in the northern, eastern, western, and southern districts of Tehran, Iran. At the time of the study, there were a total of 140 EMS stations in these four districts. Sampling was performed in several rounds. During each round, we randomly selected one station from each district and then one EMS staffs from the selected station. The inclusion criteria were having the desire for participating in the study, having an at least five-year work experience, and holding college diploma in either a two-year course of Medical Emergency or a four-year Nursing course. Finally, eighteen EMS staffs were recruited to the study by using the purposive sampling technique.

### 2.3. Data Collection

We collected the study data by conducting semistructured personal interviews. Primarily, the interview questions were framed as follows.

- How do you identify that a medical patient needs transportation?
- What do medical patients and their families expect from you?
- How do medical patients and their families react to your decisions about patient transportation to medical facilities?
- What are your criteria for transportation?

Moreover, we employed probing questions for acquiring detailed information about experiences shared by the study participants. Data collection was pursued until reaching data saturation. The sixteenth interview yielded no further helpful information or insights. However, to ensure data saturation, we conducted two more interviews. Accordingly, we conducted eighteen interviews in total. Interviews were scheduled according to participants' preferences. All participants were inclined toward being interviewed at their station and at the beginning of their working shift. Interviews lasted for 23–41 minutes (36 minutes in average). We recorded all the interviews by using a digital sound recorder. Immediately after each interview, we transcribed the interview content verbatim.

### 2.4. Data Analysis

Data analysis was carried out concurrently with data collection. We employed the qualitative content analysis approach [14] for data analysis. Whole interviews were considered as the unit of analysis. Accordingly, we read each interview several times to achieve a general understanding about it. Then, we started to code the meaning units, that is, words, sentences, or paragraphs, by using either participants' own expressions or our constructed codes. Simultaneously, codes were compared with each other and also with the new ones and categorized according to their differences and similarities. Categories were in turn compared and categorized into a higher-level overarching theme.

### 2.5. Rigor

The credibility of the study findings was established by using the member- and the peer-checking techniques [15]. Accordingly, we provided several participants with their own interview transcripts as well as our generated codes and asked them to determine whether our generated codes and concepts reflected their experiences or not. In case of any incongruence between the participants' shared experiences and our generated codes, we revised the codes based on their comments through negotiation. Moreover, we invited two qualitative content analysts to check the accuracy and appropriateness of our codes.

### 2.6. Ethical Considerations

The Institutional Review Board and the Ethics Committee of Tehran Medical University of Medical Sciences approved the study. We provided the study participants with information about the aim and the process of the study and asked them to read and sign the study informed consent form. All of the invited participants gave written informed consent for participation.

## 3. Findings

In total, eighteen male EMS staffs with an age range of 28–39 years and a mean of work experience of 6.61 years participated in the study.

The main theme of the study was the “Degree of perceived risk to staffs” selves and to patient's. In other words, the degree of the perceived risk of the emergency situation to staffs' selves and their patient determined whether a certain medical patient really needs transportation or not. This theme implied that when faced with making decision about whether to transport a patient to medical facilities or not, our participating EMS staffs considered all factors that might pose potential or real risk to them and also to their patient. This theme consisted of two main categories including “patient's condition” and “the context of the EMS mission” (Table 1). In what follows, we explain the main theme and the main categories of the study.

### 3.1. Patient's Condition

One of the most important factors affecting our participants' decision about whether to transport a patient or not was patient's condition. This category comprised three subcategories including physical health status, socioeconomic status, and cultural background.

### 3.2. Physical Health Status

Our participants mentioned that the presence of a serious disease or obvious acute signs and symptoms directly helps them decide about transporting a patient.

*“When you are certain that your patient has developed an MI (myocardial infarction), transportation is absolutely essential” (Participant 14).*

They also noted that patient's healthy physical state as well as the absence of a serious disease or obvious acute signs and symptoms made them suspicious about a real need for transportation.

*“When patient is conscious and has normal vital signs, you feel unsure about transportation” (Participant 10).*

*“When you cannot find anything abnormal in patient's body and only he says that he is not feeling good, you remain doubtful about what you should do” (Participant 1). *

### 3.3. Socioeconomic Status

Patient's socioeconomic status was another factor contributing to our participants' decision about transportation. This subcategory consisted of three main concepts including patient's support system as well as patient and his family's educational and financial status.

#### 3.3.1. Patient's Support System

The strength of patient's support system—including legal support, family, and neighbors support as well as insurance coverage—also affected our participants' decision about transportation. Legal support included health-related rules and regulations that in most cases safeguard patients' not care providers' rights. Moreover, to make sure of their patient's health, some families obliged our participants, either kindly or forcefully, to transport their patient to medical facilities. Patient's health insurance coverage was also an important factor contributing to families' insistence upon transportation.

*“When patient's family ask and insist on transportation, you have no option but to transport him even if he has no serious problem” (Participant 11).*

Another aspect of family support was related to EMS staffs' judgment about family members' ability to re-call for ambulance and successfully deliver care to their patient until ambulance arrives.

*“When you feel that patient's family members can care for him in case of developing any problem, you are more comfortable about deciding not to transport him” (Participant 16).*

#### 3.3.2. Patient and His Family's Educational Status

Another subcategory of the socioeconomic status main category was patient and his family's educational status. This factor both facilitated and hindered our participants' ability to make a right decision about patient transportation. Having higher educational status as well as having health-related education facilitated patients and their families' understanding of EMS staffs' duties, encouraged them to provide staffs with accurate information about patient's condition, and helped them respect staffs' decisions.

*“Communicating and interacting with people who have higher educational status is relatively easier” (Participant 10).*

However, in some instances, patients and family members who had higher educational status tended to hold higher expectations. Moreover, patients and family members who held health-related degrees, sometimes, meddled in EMS staffs' affairs and negatively affected their decisions. On the other hand, regarding patients and family members who had low educational status, factors such as misunderstandings and misconceptions about EMS staffs' duties, inability to establish effective communication with staffs, inability to recall patient's medical history, and indifference towards the importance of keeping patient's medical records brought about difficulties for our participants in making rational transportation-related decisions.

*“People who hold health-related degrees tend to meddle in our affairs. Then, it is hard to arrive at a right decision” (Participant 8). *

*“You cannot judge about patients and their family members based on their degrees. Some people who hold higher degrees have higher expectations while some of them who have low educational status barely understand you” (Participant 13).*

#### 3.3.3. Patient and His Family's Financial Status

Another factor affecting our participants' decision about transportation was patient and his family's financial status. According to our participants, patients and families with better financial status sometimes hold higher expectations and show higher sensitivity to their health.

*“Those who have better financial status have higher expectations. For example, (we are sure that) their patient has no problem. However, they call for ambulance and despite our advice, insist on transportation to hospital” (Participant 2). *

On the other hand, patients and families who had lower financial status were more willing to receive care at home. Moreover, if transportation was needed, they liked their patient to be transported to local clinics located in their own neighborhood. The reason was that they could not afford their own subsequent public transportation costs needed for referring to hospital for visitation.

*“The families who have financial problems and do not have insurance ask us to manage their patient's problem at home” (Participant 7). *

### 3.4. Cultural Background

The third subcategory of the patient's condition main category was patient and his family's cultural background. This subcategory comprised two main concepts including confidence as well as beliefs and attitudes.

#### 3.4.1. Confidence

Confidence affected our participants' transportation-related decisions in several ways. Our participants noted that patients and families who trust in EMS staffs' abilities are more cooperative, provide more accurate information, and respect EMS staffs' views and decisions. Conversely, lack of confidence could result in having reluctance to provide information about patient's past medical history and present illness.

*“Some patients think that if they play ill, we will transport them to hospital” (Participant 12).*

*“We also had patients who told us that in previous episodes of the same disease, they had referred to hospital and they (hospital staffs) hospitalized them” (Participant 9).*

#### 3.4.2. Beliefs and Attitudes

Our participants also noted that patients and families' beliefs and attitudes significantly contribute to their tendency to abide by EMS staffs' decisions. Previous first- or second-hand experiences of transportation as well as misconceptions and superstitions about the fate of patients who are transported by ambulance significantly affect patients and families' beliefs and attitudes about the efficiency and safety of ambulance services.

*“There was a patient who strongly insisted on transportation. He said that last year his colleague developed the same problem and ambulance didn't transport him to hospital and hence, he experienced a heart attack several hours later” (Participant 9). *

*“We had a patient who was scared of getting into ambulance because his mother had died after being transported to medical facility by ambulance” (Participant 3).*

### 3.5. The Context of the EMS Mission

The second main category of the study was the context of the EMS mission. This category consisted of two subcategories including the characteristics of the mission and EMS staffs' characteristics. These subcategories with the corresponding concepts are discussed below.

### 3.6. Characteristics of the Mission

Our participants noted that although most of the EMS missions follow a similar consistent pattern, the conditions and the characteristics of each mission are unique and differ from other missions. This subcategory comprised four main concepts including response time, possibility of obtaining advice, equipment, and special conditions.

#### 3.6.1. Response Time

According to our participants, response or arrival time is a major challenge of emergency care delivery. Late arrival, irrespective of its cause, presents EMS staffs with difficulties in deciding on transportation. In such circumstances, the most important factor that might have affected EMS staffs' decision was the unfavorable emotional atmosphere of the emergency situation. Accordingly, in case of late arrival, EMS staffs usually decided to transport patient to alleviate the condition and lighten the atmosphere.

*“Some emergency locations are remote. Sometimes, alleys have no sign and hence, patient's location is difficult to find. Accordingly, we arrive late. Sometimes, we arrive soon but the alley is too narrow or patient is located at the tenth floor and the elevator is out of order. When we finally arrive at the destination, both patient and family members are filled with intense anger with us. You are also both tired and angry. In such situations, can we make a right decision?” (Participant 6).*

#### 3.6.2. Possibility of Obtaining Advice

Our participants noted that for deciding on transportation, EMS staffs sometimes need to obtain advice from different sources including emergency physician attending at the dispatching center, colleagues attending at the emergency situation, as well as patients and their family members. An important factor contributing to the usefulness of a piece of advice was its applicability. For example, novice physicians usually recommended EMS staff to transport patients while experienced physicians provided constructive advice.

*“Sometimes, our emergency physician is novice and has not yet experienced even an EMS mission. “Transport the patient” is the only advice given by such physician” (Participant 8). *

Our participants also highlighted that the availability of an efficient wireless communication device, the ability of EMS staffs in accurately assessing the emergency situation and effectively transferring assessment data to the attending emergency physician, and physicians' ability in providing constructive advice were the important factors contributing to the possibility of obtaining advice. Other EMS colleagues were also good sources of information. Our participants mentioned that an educated, experienced EMS staff can provide other staffs with constructive advice. Moreover, patients and family members can also provide invaluable information and excellent advice in case of being communicated effectively.

*“You may call the emergency center to consult with the attending physician. However, the physician is, sometimes, so busy that you prefer to decide about transportation by seeking help and advice from your own colleague and the patient” (Participant 4). *

#### 3.6.3. Equipment

The type and the accessibility of medical equipment also were among the other factors affecting our participants' decision about transportation. They reported that inaccessibility or defectiveness of the essential equipment, such as stethoscope, sphygmomanometer, pulse oximeter, electrocardiogram machine, glucometer, and so forth, make them suspicious about assessment data, and, therefore, compel them to transport patient to medical facilities for further assessment. Conversely, availability of basic high-tech equipment helped them make more accurate decisions.

*“Since we have been equipped with portable glucometers, we have fewer difficulties in making decision about transporting diabetic patients. However, some glucometers are defective and accordingly, we cannot diagnose whether patients' weakness is related to his low blood glucose level or not. In such instances, we feel compelled to transport the patient” (Participant 9).*

#### 3.6.4. Special Cases

According to our participants, some of the EMS missions are performed for providing care to special cases such as patients who hold strategic management or administrative positions, elderly people who live alone and call for ambulance at midnight, students who develop problems at school, culprits and prisoners, and foreigners. They noted that in these cases, they have to transport the patient irrespective of the severity or the seriousness of the problem.

*“For example, in case of confronting a culprit in a police station who is holding his belly, shouting that ‘I'm having pain', we are left with no option but transport him even if he is shamming. Otherwise, if he develops any problem later, they will accuse us of negligence and malpractice” (Participant 5). *

*“When they call ambulance for a (foreign) tourist, we immediately transport him. Because, if we do not transport him and something wrong happens to him, our hosting country would get into trouble” (Participant 9). *

### 3.7. EMS Staffs' Characteristics

Personal characteristics of EMS staffs as well as their working environment were also key factors in transportation-related decisions. This subcategory consisted of three main concepts including reasoning ability, physical health status, and perceived support.

#### 3.7.1. Reasoning Ability

EMS staffs' reasoning—developed over time through gaining knowledge and experience—also could affect their transportation-related decisions. Our participants noted that compared with obvious injuries and traumas, diagnosing medical problems is much more difficult. Accordingly, EMS staffs who have more knowledge and experience have better reasoning ability and hence reach sensible decisions more easily and more quickly.

*“Since participating in a workshop on acute heart problems, I have become more sensitive to the manifestations of heart problems” (Participant 15).*

*“We have had so many medical patients that now we can diagnose medical problems easily” (Participant 17).*

#### 3.7.2. Physical Health Status

EMS staffs' physical condition also affected their transportation-related decisions. According to our participants, physical problems such as fatigue, sleepiness, the flu, headache, and musculoskeletal pain may negatively affect EMS staffs' concentration, resulting in poor decisions and subsequently serious consequences.

*“My colleague was awfully tired and hence failed to diagnose a true myocardial infarction. He transferred the afflicted patient from the fourth floor to ambulance without using a stretcher. Accordingly, patient's condition deteriorated” (Participant 1).*

*“When you are on a long 24-hour shift, you are no longer in mood for assessing and talking to patient. Instead, you transport all patients to avoid getting into trouble later on” (Participant 18). *

#### 3.7.3. Perceived Support

The strength of EMS staffs' support system—including legal, organizational, professional, managerial, and financial support as well as liability insurance coverage—was also a key factor affecting our participants' decision about patient transportation. EMS technicians who did not have an effective support system made decisions that carried minimal risk.

*“When your senior is looking for an opportunity to pick on you for your faults and on the other hand, patient's family members insist on transportation, you prefer to transport the patient. Otherwise, if anything wrong happens, you will be alone (i.e. the senior will not support you)” (Participant 11).*

## 4. Discussion

The aim of this study was to explore factors affecting EMS staffs' decision about transporting medical patients to medical facilities. Study findings revealed that the main factor affecting our participants' transportation-related decisions was the degree of the perceived risk of the emergency situation to staffs' selves and their patient. Other factors such as patient's physical condition, socioeconomic status, cultural background, characteristics of the mission, and EMS staffs' characteristics contributed to the abovementioned main factor. We discuss these factors below.

Patients' physical health status was a contributing factor affecting our participants' decision about transportation. For example, patients' vital signs are among the main criteria for decision making. Generally, vital signs are the key components of prehospital assessment checklist. Duckitt et al. noted that physiological parameters such as heart rate, systolic blood pressure, body temperature, oxygen saturation, and level of consciousness are among the most important factors determining the need for medical admission [16]. Rees and Mann also used physiological parameters such as central nervous system response, respiratory rate, heart rate, and systolic blood pressure for identifying high-risk patients in emergency department [17]. Our findings also revealed that besides the absence of normal physiological parameters, the presence of pathologic conditions also played an important role in deciding on transportation. For example, if our participants noticed a life-threatening condition (such as an acute myocardial infarction), they immediately decided to transport the patient irrespective of other parameters and conditions. The important fact here is that the signs and the symptoms of pathologic conditions are not necessarily obvious and hence EMS staffs require a great amount of knowledge and expertise for diagnosing them. Consequently, EMS staffs need education to be empowered enough for easily and correctly diagnosing medical problems. Nonetheless, Frost and Wise noted that even if the underlying disease has not yet been diagnosed, life-threatening conditions—such as coma, convulsion, restlessness and confusion, tachycardia, bradycardia, decreased blood pressure, coldness, cyanosis, tachypnea, bradypnea, and anuria—are easily identifiable [18].

Patients' socioeconomic status and cultural background were the other factors affecting our participants' decision about transportation. Other studies have also shown that socioeconomic status is a determinant of health [19–21] and lack of resources and facilities makes patients and families vulnerable [22]. However, these parameters are not routinely used for making standard clinical judgments. These findings indicate that prehospital decision making is not performed solely based on clinical judgment criteria.

We also found that the characteristics of EMS mission—including response time, possibility of obtaining advice, equipment, and special conditions—also affected our participants' decision about transportation. The response time (the time between patient's call for ambulance and the arrival of ambulance) is a determining factor in decreasing the risk of life-threatening complications and improving survival [23, 24]. In large cities like Tehran, the response time is below the international standards—14.98 [25] versus less than eight minutes [26]. Possibility of obtaining advice also contributed to our participants' decision about transportation. In different complicated situations, our participants tended to get advice from different sources such as the attending emergency physician, other EMS staffs, as well as patients and their family members. If our participants could obtain constructive advice, they could make better decisions about transportation. Otherwise, they felt doubt over the best decision and, in most cases, finally decided to transport the patient irrespective of the real need for transportation. McCaughan et al. also found that in awkward situations, nurses usually preferred to get advice from physicians and their own colleagues [27]. Defectiveness or inaccessibility of medical equipment were another factor affecting our participants' decision about transportation. We found that equipment malfunction required our participants to quickly decide on transportation. Assar-roudi also reported that shortage of medical equipment interferes with efficient emergency care delivery [28]. Moreover, the study findings revealed that in special cases, EMS staffs needed to take into account different political or security considerations when deciding on transportation. This finding indicates that EMS staffs are under uncontrollable external pressures that negatively affect their decisions and professional practice.

Another factor affecting our participants' decision about transportation was their own characteristics—including their reasoning ability, physical health status, and perceived support. We found that our participating staffs integrated their knowledge and expertise to better understand patients' conditions and hence make more sensible decisions. Integration of knowledge and expertise—which is sometimes referred to as intuition—helps clinicians make important clinical decisions [29, 30]. EMS staffs' physical condition also affected their decisions about transportation. Poor physical health negatively affected their concentration as well as their relationship with patients and family members. West et al. reported that care providers' physical disorders led to problems in establishing relationship with their clients and providing care to them [31]. Ozyurt et al. also noted that fatigue negatively affects workers' professional commitment and performance as well as their practical effectiveness [32].

The level of perceived socioeconomic and organizational support also affected our participants' decisions about transportation. When they perceived stronger support, they could make decisions that were more sensible. According to Taylor, there are different types of support available to individuals—appraisal support, financial support, informational support, and emotional support [33]. Rosenfeld et al. noted that weak social support creates distrust and uncertainty, which in turn might result in rule violation [34]. Accordingly, inadequate support perceived by EMS staffs may result in the violation of transportation rules. We found that EMS staffs who perceived weak support tended to transport all medical patients, irrespective of their real need for transportation. However, in case of lack of support, there is also a potential risk of refraining from transporting patients who really need transportation.

## 5. Conclusion

Study findings suggest that many factors—with different degrees of importance—contribute to EMS staffs' decision about patients' need for transportation. The multiplicity of these factors implies the tremendous responsibility of EMS staffs in accurately diagnosing medical problems. They need to analyze these factors in a short period of time and finally reach a right decision about transportation. Consequently, EMS staffs should be recruited from well-educated highly-experienced healthcare professionals. Moreover, EMS system managers can help facilitate their staffs' decision making and lighten their workload through providing them with adequate technical, informational, financial, educational, and emotional support. The study findings also provide EMS staffs with considerable support against criticism about EMS staffs' judgments and decisions being non-evidence-based.

Given the diversity and the multiplicity of factors that affect EMS staffs' decision about patient transportation, the development of effective, user-friendly checklists and scoring systems for quickly and easily identifying medical patients' need for transportation is recommended. Moreover, as other factors may contribute to EMS patient transportation decision, replicating this study in other contexts and settings is also recommended.

## 6. Limitations of the Study

We strived to create a comfortable and supportive environment during the interviews. Nonetheless, some of the participants might have taken into account different personal and organizational considerations when sharing their experiences.

## Figures and Tables

**Table 1:** Affecting factors of emergency medical services staffs' decision about transporting medical patients to medical facilities.

Main theme: degree of perceived risk to staffs' selves and to patients		
Categories	Subcategories	Concepts
Patient's condition	Physical health status	Presence of normal physiological parameters; the presence of pathologic conditions
		Absence of normal physiological parameters
		Presence of an obvious, serious disease
	Socioeconomic status	*Patient's support system *
		*Patient and his family's educational status *
		*Patient and his family's financial status *
	Cultural background	*Confidence *
		*Beliefs and attitudes *

The context of the EMS mission	Characteristics of the mission	*Response time *
		*Possibility of obtaining advice *
		*Equipment *
		*Special cases *
	EMS staffs' characteristics	*Reasoning ability *
		*Physical health status *
		*Perceived support *
